# The Etiology of Pneumonia From Analysis of Lung Aspirate and Pleural Fluid Samples: Findings From the Pneumonia Etiology Research for Child Health (PERCH) Study

**DOI:** 10.1093/cid/ciaa1032

**Published:** 2020-07-25

**Authors:** Bernard E Ebruke, Maria Deloria Knoll, Meredith Haddix, Syed M A Zaman, Christine Prosperi, Daniel R Feikin, Laura L Hammitt, Orin S Levine, Katherine L O’Brien, David R Murdoch, W Abdullah Brooks, J Anthony G Scott, Karen L Kotloff, Shabir A Madhi, Donald M Thea, Vicky L Baillie, Mohammod Jobayer Chisti, Michel Dione, Amanda J Driscoll, Nicholas Fancourt, Ruth A Karron, Tham T Le, Shebe Mohamed, David P Moore, Susan C Morpeth, John Mwaba, James Mwansa, Abu Sadat Mohammad Sayeem Bin Shahid, Samba O Sow, Milagritos D Tapia, Martin Antonio, Stephen R C Howie

**Affiliations:** 1 Medical Research Council Unit, Basse, The Gambia; 2 International Foundation Against Infectious Disease in Nigeria (IFAIN), Herbert Macaulay Way Central Business District, Abuja, Nigeria; 3 Department of Pediatrics, College of Medicine, University of Nebraska Medical Center, Omaha, Nebraska, USA; 4 Department of International Health, International Vaccine Access Center, Johns Hopkins Bloomberg School of Public Health, Baltimore, Maryland, USA; 5 London School of Hygiene and Tropical Medicine, London, United Kingdom; 6 Liverpool School of Tropical Medicine, Liverpool, United Kingdom; 7 Kenya Medical Research Institute–Wellcome Trust Research Programme, Kilifi, Kenya; 8 Department of Pathology, University of Otago, Christchurch, New Zealand; 9 Microbiology Unit, Canterbury Health Laboratories, Christchurch, New Zealand; 10 Department of International Health, Johns Hopkins Bloomberg School of Public Health, Baltimore, Maryland, USA; 11 International Centre for Diarrhoeal Disease Research, Bangladesh, Dhaka, Bangladesh; 12 Department of Infectious Disease Epidemiology, London School of Hygiene and Tropical Medicine, London, United Kingdom; 13 Department of Pediatrics, Center for Vaccine Development and Global Health, University of Maryland School of Medicine, Baltimore, Maryland, USA; 14 Medical Research Council, Respiratory and Meningeal Pathogens Research Unit, University of the Witwatersrand, Johannesburg, South Africa; 15 Department of Science and Technology/National Research Foundation, Vaccine Preventable Diseases Unit, University of the Witwatersrand, Johannesburg, South Africa; 16 Department of Global Health, Boston University School of Public Health, Boston, Massachusetts, USA; 17 Dhaka Hospital, Nutrition and Clinical Services Division, International Centre for Diarrhoeal Disease Research, Bangladesh, Dhaka, Bangladesh; 18 International Livestock Research Institute, Ouagadougou, Burkina Faso; 19 Royal Darwin Hospital, Darwin, Australia; 20 Department of International Health, Center for Immunization Research, Johns Hopkins Bloomberg School of Public Health, Baltimore, Maryland, USA; 21 Department of Pharmaceutical Health Services Research, University of Maryland, Baltimore, Maryland, USA; 22 Department of Paediatrics and Child Health, Chris Hani Baragwanath Academic Hospital and University of the Witwatersrand, Johannesburg, South Africa; 23 Microbiology Laboratory, Middlemore Hospital, Counties Manukau District Health Board, Auckland, New Zealand; 24 Department of Pathology and Microbiology, University Teaching Hospital, Lusaka, Zambia; 25 Zambia Center for Applied Health Research and Development, Lusaka, Zambia; 26 Department of Microbiology, Lusaka Apex Medical University, Lusaka, Zambia; 27 Nutrition and Clinical Services Division, International Centre for Diarrhoeal Disease Research, Bangladesh, Dhaka, Bangladesh; 28 Centre pour le Développement des Vaccins, Bamako, Mali; 29 Department of Pathogen Molecular Biology, London School of Hygiene and Tropical Medicine, London, United Kingdom; 30 Microbiology and Infection Unit, Warwick Medical School, University of Warwick, Coventry, United Kingdom; 31 Department of Paediatrics, University of Auckland, Auckland, New Zealand

**Keywords:** lung aspirate, pleural fluid aspirate, PERCH, pneumonia, etiology childhood

## Abstract

**Background:**

An improved understanding of childhood pneumonia etiology is required to inform prevention and treatment strategies. Lung aspiration is the gold standard specimen for pneumonia diagnostics. We report findings from analyses of lung and pleural aspirates collected in the Pneumonia Etiology Research for Child Health (PERCH) study.

**Methods:**

The PERCH study enrolled children aged 1–59 months hospitalized with World Health Organization–defined severe or very severe pneumonia in 7 countries in Africa and Asia. Percutaneous transthoracic lung aspiration (LA) and pleural fluid (PF) aspiration was performed on a sample of pneumonia cases with radiological consolidation and/or PF in 4 countries. Venous blood and nasopharyngeal/oropharyngeal swabs were collected from all cases. Multiplex quantitative polymerase chain reaction (PCR) and routine microbiologic culture were applied to clinical specimens.

**Results:**

Of 44 LAs performed within 3 days of admission on 622 eligible cases, 13 (30%) had a pathogen identified by either culture (5/44) or by PCR (11/29). A pathogen was identified in 12/14 (86%) PF specimens tested by either culture (9/14) or PCR (9/11). Bacterial pathogens were identified more frequently than viruses. All but 1 of the cases with a virus identified were coinfected with bacterial pathogens. *Streptococcus pneumoniae* (9/44 [20%]) and *Staphylococcus aureus* (7/14 [50%]) were the predominant pathogens identified in LA and PF, respectively.

**Conclusions:**

Bacterial pathogens predominated in this selected subgroup of PERCH participants drawn from those with radiological consolidation or PF, with *S. pneumoniae* and *S. aureus* the leading pathogens identified.

## Teaser

Lung aspiration (LA) and pleural fluid (PF) aspiration were performed on a subgroup of PERCH pneumonia cases with radiological consolidation or PF collection. Bacterial pathogens predominated (*Streptococcus pneumoniae* in LA, *Staphylococcus aureus* in PF), in contrast to overall PERCH findings.

Pneumonia remains a leading cause of child mortality, with the overwhelming burden of deaths occurring in developing countries [1]. An improved understanding of childhood pneumonia etiology is required to inform prevention and treatment strategies.

The Pneumonia Etiology Research for Child Health (PERCH) study aimed to identify the causes and risk factors for severe childhood pneumonia in developing countries in the pneumococcal and *Haemophilus influenzae* type b (Hib) conjugate vaccine era, and is the largest such study since the 1980s [2, 3].

PERCH applied conventional microbiological and molecular diagnostic techniques to a wide range of clinical specimens including percutaneous transthoracic fine needle lung aspirates (LA) and pleural fluid (PF) aspirates, which obtain a specimen directly from the site of infection. Such samples, though rarely obtained in practice, together with the application of molecular diagnostic techniques, have been shown to significantly improve the diagnostic yield in pneumonia etiology studies [4, 5]. This article reports findings from LA and PF specimens collected in the PERCH study.

## METHODS

PERCH was a multicountry case control study of severe or very severe pneumonia etiology carried out in 9 sites in 7 participating countries (The Gambia, Mali, Kenya, Zambia, South Africa, Bangladesh, and Thailand) [6, 7]. Cases were children aged 1–59 months with World Health Organization (WHO)–defined severe or very severe pneumonia (pre-2013 definition) [6]. Children with wheeze whose lower chest wall indrawing resolved following bronchodilator therapy were excluded. Each site recruited participants for 2 years between August 2011 and January 2014. Case enrollment, specimen collection, and laboratory procedures were standardized [7–10]. Percutaneous transthoracic lung aspiration was performed on eligible cases in the 4 PERCH countries where ethical approval for LA collection was obtained: The Gambia, South Africa, Mali, and Bangladesh. In The Gambia, lung aspirations were done throughout the case enrollment period, but at the 3 other sites the procedure commenced at various times into the recruitment period based on when ethical approvals were obtained and staff were trained in specimen collection techniques. PF was collected at all sites per local clinical practice guidelines. Pneumococcal conjugate vaccine (PCV) had been introduced prior to the study in Kenya (10-valent), The Gambia, Mali, and South Africa (13-valent [PCV13]).

### Ethical Considerations

Ethical approval for the PERCH study, and for the use of fine needle lung aspiration procedure, was obtained from the Johns Hopkins Bloomberg School of Public Health Institutional Review Board and from local ethics review boards for each participating site. Written informed consent was obtained from guardians for all procedures undertaken.

### Lung Aspiration

Fine needle percutaneous lung aspiration was used in this study, the details of which are described, and it is important not to confuse this with percutaneous lung biopsy, which has a materially different safety profile.

#### Site Preparation

A standardized protocol for specimen collection procedures was developed from The Gambia site, which has a long history of safety and utility of the procedure [11]. Training and subsequent support for clinical staff at the other participating PERCH sites were provided by experienced clinicians from The Gambia.

#### Case Eligibility

Cases were eligible for lung aspiration if they had a peripheral confluent alveolar consolidation identified on chest radiograph (CXR) by the treating clinician, no contraindications (eg, presence of pneumatoceles on CXR and postmeasles pneumonia), and written informed consent. The procedure was deferred in children who were clinically unstable. Ultimately, the decision to undertake lung aspiration on eligible children rested with the attending clinician and consent by the child’s parents/legal guardians.

#### Procedural Steps

As described elsewhere [11], children were positioned sitting or supine and the site of consolidation seen on CXR was identified clinically by locating an area of maximal dullness to percussion or crepitations on auscultation. Skin over the identified area was sterilized. A standard 21-gauge hypodermic needle attached to a 5-mL syringe into which 1 mL of sterile 0.9% saline had been aspirated was used for the procedure. The needle was inserted over the superior aspect of the rib and advanced into the identified area of maximal dullness on percussion, strictly avoiding the cardiac area. The area was then aspirated immediately and the needle withdrawn, over about 2 seconds, maintaining maximal suction pressure on the syringe. With the needle used for the lung aspiration procedure still attached, aspirate contents were flushed into a sterile universal specimen container.

#### Safety Monitoring

Safety monitoring for the LA procedure included close observation of cases including vital signs and oximetry for at least 4 hours postprocedure. Adverse events were defined according to the protocol and reported to the study safety monitor.

### Pleural Fluid and Other Clinical Specimens

Cases with pleural fluid on CXR and/or clinical assessment were considered for aspiration of PF, which was done according to routine clinical practice [12, 13]; however, this assessment was not standardized across sites. Treating clinicians interpreted CXRs for PF specimen eligibility in real time.

Other specimens including venous blood and nasopharyngeal/oropharyngeal (NP/OP) swabs were collected using standardized methods described previously [9, 10].

### Laboratory Methods

Laboratory testing methods used for these analyses have been described separately [10, 14–17]. In brief, we used multiplex quantitative polymerase chain reaction (PCR) (FTD Resp-33 kit; Fast-track Diagnostics, Sliema, Malta) and routine culture to test LA, PF, and NP/OP specimens. Blood was cultured for bacterial pathogens and was also tested for *lyt*A (a molecular marker of *S. pneumoniae*) by PCR. Pneumococcal serotypes were determined by Quellung method or PCR; microarray was used to determine serotype for NP/OP-culture negative but PCR-positive specimens [18]. For *H. influenzae*, all culture isolates were serotyped; the FTD panel included an all-serotype *H. influenzae* PCR target and an Hib target.

### Statistical Analysis

For both LA and PF, only those specimens obtained within 3 days of enrollment were included in analyses to limit the potential impact of nosocomial infection on findings. A PERCH reading panel provided standardized CXR interpretations independent of clinical care [19]. Selected baseline characteristics were compared between cases with LA and all other cases categorized as having consolidation on CXR as per the PERCH CXR reading panel, restricting to the 4 sites performing lung aspiration [19]. Categorical variables were assessed using logistic regression (adjusted for age and site, where relevant), and the Kruskal-Wallis test was performed for continuous variables. For cases with LA or PF specimens, frequencies and proportions of cases with pathogens identified by culture and by PCR were calculated, restricting analyses to those children with results available for the measurement. We assessed concordance in the pathogens detected between LA and PF specimens compared to NP/OP and blood culture specimens.

## RESULTS

### Lung Aspirates

**Figure 1.**
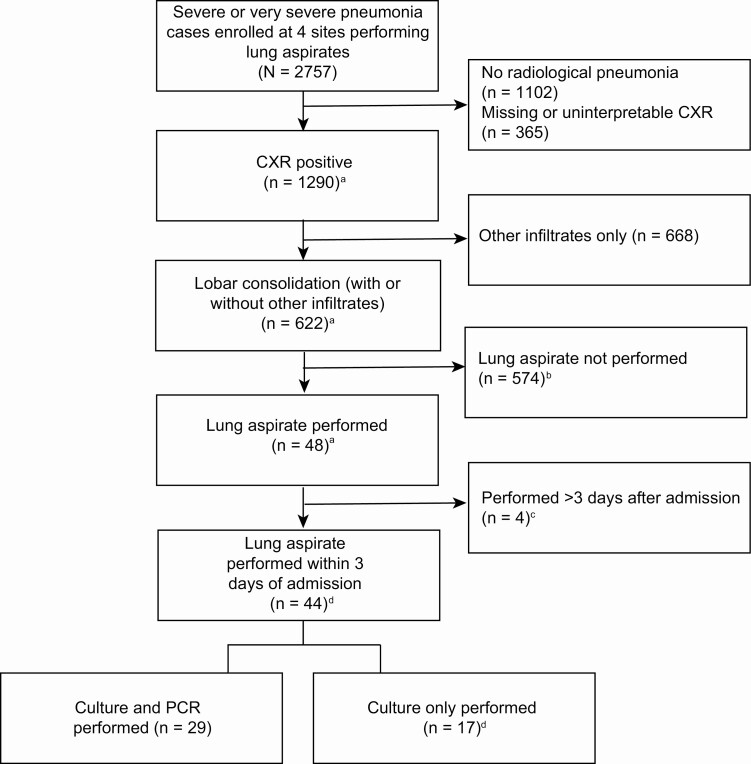
Enrollment of lung aspiration cases (n = 44) in 4 Pneumonia Etiology Research for Child Health (PERCH) sites performing lung aspiration. ^a^Includes 2 cases the PERCH chest radiograph (CXR) reading panel determined to have normal CXR results and 2 cases the panel determined to have uninterpretable results. The decision to perform the procedure was made by the clinical team responsible for care of the patient, based on information available to it at the time of admission. ^b^Lung aspirates not performed due to procedure not yet initiated at site or eligible but not done. ^c^Includes 1 case that the PERCH CXR reading panel determined to have normal CXR results. ^d^Includes 1 case that the PERCH CXR reading panel determined to have normal CXR results, and 2 cases that the panel determined to have uninterpretable results. Abbreviations: CXR, chest radiograph; PCR, polymerase chain reaction.

Of 2757 children enrolled at the 4 sites performing lung aspiration, 622 (23%) had CXR confluent alveolar consolidation, of whom 48 (8%) had LA performed (Figure 1). Forty-four of the 48 (92%) lung aspirations were performed within 3 days of enrollment (61% on the day of admission) and are included in these analyses. One of the 44 cases had CXR findings judged to be normal by the CXR reading panel and 2 had uninterpretable CXRs; the remaining 41 cases had a CXR finding of consolidation confirmed by the panel. The main reasons for ineligibility for LA were nonconsent by the parent/caregiver and that in the attending clinician’s judgment the procedure was not safe to perform, generally because of the location of consolidation or the child’s clinical condition. Failure to collect a LA sample from a potentially eligible case was either because the site had not yet had training in the procedure or there were operational barriers that prevented it, such as high workloads and logistical challenges with the LA specimen collection during after-hours or weekends.

LA cases were similar to all other pneumonia cases with CXR consolidation but in whom an LA specimen was not taken with respect to clinical and demographic characteristics such as the median age, pneumonia severity, vaccination status, and median duration of hospital stay, but not by site, the majority (24/44 [54.5%]) of lung aspirations being performed at The Gambia site (Table 1). The proportion of cases pretreated with antibiotics prior to LA collection was lowest in The Gambia (9/24 [38%]) and was 80% or higher in all other sites (Supplementary Table 1). Cases where a pathogen was detected on LA (culture or PCR) had a higher prevalence of fever (69% vs 25%; *P* < .001) and lethargy (31% vs 8%; *P* = .02) and an absence of wheezing (0% vs 27%; *P* = .02) compared to all other cases with CXR consolidation but in whom no LA sample was obtained (Supplementary Table 2).

**Table 1.** Comparison of Clinical and Demographic Characteristics of Cases With Consolidation on Chest Radiograph Who Did and Did Not Have Lung Aspirates Collected (Adjusted for Age and Site^a^)

Characteristic	Cases With LA Collected (n = 44)	Cases With No LA Collected (n = 574)	*P* Value^a^
Age, mo, median (IQR)	8.5 (3–19)	7 (3–13)	.18
Age <1 y	26 (59)	396 (69)	.39
Female sex	20 (46)	284 (50)	.69
Site			
The Gambia	24 (54)	81 (14)	<.0001
Mali	10 (23)	135 (24)	
South Africa	6 (14)	304 (53)	
Bangladesh	4 (9)	54 (9)	
Very severe pneumonia (vs severe)	14 (32)	187 (33)	.27
Duration of illness, d, median (IQR)^b^	3.5 (3–6)	3 (2–6)	.90
Hypoxemia^c^	14 (32)	321 (56)	.93
Tachycardia^d^	26 (59)	293 (51)	.51
Temperature ≥38°C	22 (50)	142 (25)	.30
Wheeze^e^	8 (18)	157 (27)	.11
Danger signs			
Head nodding	6 (14)	146 (25)	.80
Central cyanosis	1 (2)	17 (3)	.77
Inability to feed/drink	2 (4.5)	41 (7)	.86
Vomiting everything	0 (0)	7 (1)	.73
Lethargy	8 (18)	43 (7.5)	.07
Multiple or prolonged convulsions	3 (7)	13 (2)	.14
Antibiotic pretreatment^f^	11 (25)	260 (45)	.34
Vaccination			
At least 1 HibCV dose	35 (83)	416 (77)	.98
At least 1 PCV dose	31 (74)	355 (70)	.29
Severe wasting^g^	6 (14)	100 (18)	.88
HIV positive	4 (9)	69 (12)	.29
Died in hospital	2 (4.5)	44 (8)	.48
RSV NP/OP PCR Positive	6 (14)	129 (23)	.85
Parainfluenza 1 NP/OP PCR Positive	3 (7)	25 (4)	.50

Data are presented as no. (%) unless otherwise indicated. Table restricted to sites where LA specimens were available (The Gambia, South Africa, Bangladesh, and Mali). Cases with LA specimens collected >72 hours after enrollment were excluded from the analysis. Consolidation was based on Pneumonia Etiology Research for Child Health (PERCH) standardized chest radiograph (CXR) reading panel, not clinician reading during hospitalization.

Abbreviations: HibCV, *Haemophilus influenzae* type b conjugate vaccine; HIV, human immunodeficiency virus; IQR, interquartile range; LA, lung aspirate; NP/OP PCR, nasopharyngeal/oropharyngeal polymerase chain reaction; PCV, pneumococcal conjugate vaccine; RSV, respiratory syncytial virus.

^a^ *P* values based on a logistic regression model adjusted for age in months and site, with Firth adjustment for categorical variables and Kruskal-Wallis test for continuous variables comparing cases with LA specimen taken to cases with consolidation on CXR but without LA specimen taken.

^b^Number of days with cough, fever, difficulty breathing, wheeze, or runny nose, whichever symptom was longest.

^c^Hypoxemic defined as oxygen saturation at admission <90% at South Africa and <92% at other sites, or oxygen requirement (if on oxygen and room air saturation not available).

^d^Elevated heart rate at baseline clinical assessment defined as follows: >160 beats per minute (bpm) in infants 0–11 months of age, >150 bpm in children 12–35 months of age, >140 bpm in children 36–59 months of age.

^e^Presence of audible or auscultatory wheeze at admission.

^f^Antibiotic pretreatment was defined as having either a positive serum bioassay or documentation of antibiotics administered at the referral or study hospital prior to NP/OP specimen collection.

^g^Weight-for-height *z* score < −3.

All LA samples had culture results available, but only 29 of 44 (66%) had PCR results available, with missing PCR results due to low specimen volume. Pathogens were identified by culture in 5 of 44 (11%) cases and by PCR in 11 of 29 (38%) cases (Table 2). Thirteen LA samples had a pathogen detected by either method (13/44 [30%]), with 7 of 13 (54%) positive for multiple pathogens (Table 2) and 9 of 13 (69%) from The Gambia (Supplementary Table 3). Pathogen detection decreased with increasing days between admission and LA collection (Supplementary Table 4). *Streptococcus pneumoniae* was the predominant pathogen (9/44 [20%]; 5/9 identified as single infections) followed by *H. influenzae* (4/44 [9%], 3 being non–type b and 1 type b) and *Moraxella catarrhalis* (4/44 [9%]). Of 4 cases with a virus identified, 2 were positive for cytomegalovirus (CMV), 1 for adenovirus, and 1 for human metapneumovirus (Table 2); most viral infections on LA (3/4 cases) were identified as coinfections with bacterial pathogens. CMV was the only virus found by itself. Of 9 cases in whom *S. pneumoniae* was identified through culture and/or PCR of LA specimens, 5 (56%) were identified as single infections (Supplementary Table 3). Six cases had >1 bacterium identified, including 2 cases with *S. pneumoniae* and *H. influenzae* coinfection, and 2 cases with *S. pneumoniae* and *M. catarrhalis* coinfection. *Pneumocystis jirovecii* was identified in 1 human immunodeficiency virus–uninfected case from The Gambia, as a coinfection with *S. pneumoniae*, *H. influenzae*, *M. catarrhalis*, and CMV (Supplementary Table 3). Among the LA cases with both culture and PCR results and who had a pathogen identified (n = 9), 3 (33%) cases had the same bacteria identified in both culture and PCR, whereas an additional 6 cases had bacteria detected in PCR only (Supplementary Table 5). Of 42 LA specimens tested for *Mycobacterium tuberculosis* by culture, none were positive.

**Table 2.** Organisms Identified by Culture and/or Polymerase Chain Reaction of Lung Aspirate Specimens

Organism	PCR (n = 29)	Culture (n = 44)	Either PCR or Culture (n = 44)
Any positive^a^	11 (38)	5 (11)	13 (30)
* Streptococcus pneumoniae*	7 (24)	5 (11)	9 (20)
* Haemophilus influenzae*	4 (14)	1 (2)	4 (9)
* Chlamydia pneumoniae*	1 (3)	0	1 (2)
* Moraxella catarrhalis*	4 (14)	0	4 (9)
* Pneumocystis jirovecii*	1 (3)	0	1 (2)
* *Adenovirus	1 (3)	NA	1 (2)
* *CMV	2 (7)	NA	2 (4)
* *HMPV	1 (3)	NA	1 (2)
Combinations^b^			
* S. pneumoniae + H. influenzae*	…	…	2 (4)
* S. pneumoniae + M. catarrhalis*	…	…	2 (4)
* *Adenovirus + *C. pneumoniae*	…	…	1 (2)
* H. influenzae + M. catarrhalis + S. pneumoniae + P. jirovecii* + CMV	…	…	1 (2)
* H. influenzae + M. catarrhalis +* HMPV	…	…	1 (2)

Data are presented as no. (%).

Abbreviations: CMV, cytomegalovirus; HMPV, human metapneumovirus; NA, not applicable; PCR, polymerase chain reaction.

^a^Total number of cases with organism identified is not the sum of the number of organisms identified because some cases tested positive for >1 organism.

^b^Combinations presented as detection by either PCR or culture, not split by detection method.

In the 13 LA-positive cases that had paired blood culture results available, 3 cases were blood culture positive and 2 of those had the same bacteria detected in both specimens (Supplementary Table 3). Of the 5 LA cultures that grew *S. pneumoniae*, 3 were PCV13-type serotypes (serotypes 1, 5, 6A) and 2 were non PCV13-type serotypes (serotypes 12F, 20), and all had received 3 doses of PCV. Four of these cases were *S. pneumoniae–*positive by NP culture and/or blood culture. There was serotype concordance in 3 of 4 of these cases (Supplementary Tables 3 and 6).

### Pleural Fluid

**Figure 2.**
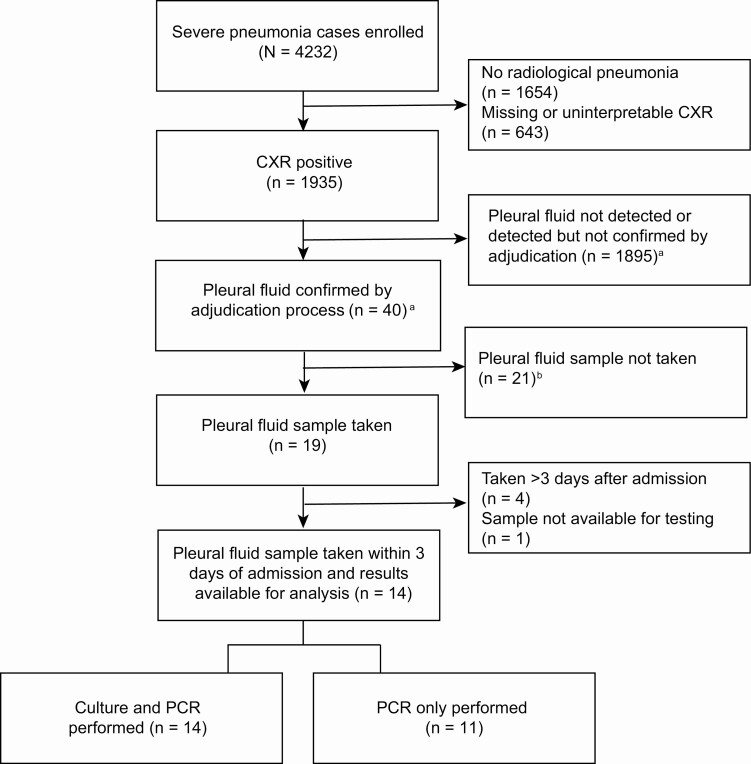
Enrollment of pleural fluid (PF) cases (n = 19) in the Pneumonia Etiology Research for Child Health (PERCH) study. ^a^Clinicians from The Gambia PERCH site reviewed all of the chest radiographs (CXRs) from those cases where ≥2 standardized readers indicated pleural effusion, or where the case had a PF specimen obtained. The cases confirmed to have presence of any PF on CXR by the clinical review team were considered confirmed by the adjudication process. See Supplementary Appendix for more details. ^b^An additional 3 PF samples were obtained but not captured here because the adjudication process determined that their radiograph did not have evidence of PF. Two were obtained from children with consolidation on CXR (sample collected on day of admission in 1 case and on day 3 postadmission in the other) and 1 with an uninterpretable radiograph (specimen collected on day 6 postadmission). Abbreviations: CXR, chest radiograph; PCR, polymerase chain reaction.

Of 19 cases with pleural fluid confirmed by the adjudication process and PF samples collected, 5 were excluded from analysis, due to collection >3 days after admission (n = 4) and unavailable test results (n = 1) (Figure 2). Cases that had PF samples were similar with respect to clinical and demographic characteristics to children who were eligible for the procedure but did not have it performed (Table 3).

**Table 3.** Clinical Characteristics of Cases With Pleural Fluid (PF)^a^ on Initial Chest Radiograph Comparing Those With PF Collected and PF Not Collected

Characteristic	Cases With PF Collected (n = 14)	Cases Without PF Collected (n = 21)	*P* Value^b^
Age, mo, median (IQR)	19 (7–36)	12 (4–27)	.17
Age <1 y	4 (29)	10 (48)	.61
Female sex	10 (71)	9 (43)	.15
Site			
Kenya	2 (14)	7 (33)	.82
The Gambia	1 (7)	3 (14)	
Mali	5 (36)	6 (29)	
Zambia	2 (14)	3 (14)	
South Africa	4 (29)	2 (10)	
HIV positive	0 (0)	1 (5)	.60
Very severe pneumonia (vs severe)	3 (21)	5 (24)	.74
Hypoxemia^c^	8 (57)	11 (52)	.79
Tachycardia^d^	9 (64)	13 (62)	.64
Temperature ≥38°C	7 (50)	14 (67)	.22
Wheeze^e^	0 (0)	3 (14)	.29
Antibiotic pretreatment^f^	8 (57)	8 (38)	.87
Vaccination			
At least 1 HibCV dose	11 (79)	15 (88)	.39
At least 1 PCV dose	8 (67)	11 (65)	.91
Severe malnutrition^g^	0 (0)	2 (10)	.61
Died in hospital	1 (7)	3 (14)	.94
RSV NP/OP PCR Positive	0 (0)	5 (25)	.11
Parainfluenza 1 NP/OP PCR Positive	0 (0)	0 (0)	

Data are presented as no. (%) unless otherwise indicated. Table restricted to sites where PF specimens were available (Kenya, The Gambia, Mali, Zambia, and South Africa); no samples were collected at the Asian sites. Cases with PF specimens collected >72 hours after enrollment were excluded from the analysis.

Abbreviations: HibCV, *Haemophilus influenzae* type b conjugate vaccine; HIV, human immunodeficiency virus; IQR, interquartile range; PCV, pneumococcal conjugate vaccine; PF, pleural fluid; NP/OP PCR, nasopharyngeal/oropharyngeal polymerase chain reaction; RSV, respiratory syncytial virus.

^a^PF identified by at least 2 readers or arbitrators in the chest radiograph (CXR) reading process on the first CXR taken, and confirmed by The Gambia Pneumonia Etiology Research for Child Health (PERCH) clinicians, regardless of CXR final conclusion based on the PERCH CXR reading panel. Only includes cases with pleural fluid seen on CXR from sites where PF specimens were taken.

^b^ *P* values based on a logistic regression model adjusted for age and site for categorical variables, and Kruskal-Wallis for continuous variables.

^c^Hypoxia defined as oxygen saturation at admission <90% at South Africa and Zambia, and <92% at all other sites, or oxygen requirement (if on oxygen and room air saturation not available).

^d^Elevated heart rate at baseline clinical assessment defined as follows: >160 beats per minute (bpm) in infants 0–11 months of age, >150 bpm in children 12–35 months of age, >140 bpm in children 36–59 months of age.

^e^Presence of audible or auscultatory wheeze at admission.

^f^Antibiotic pretreatment was defined as having either a positive serum bioassay or documentation of antibiotics administered at the referral or study hospital prior to NP/OP specimen collection.

^g^Weight-for-height *z* score < −3.

A pathogen was identified in 12 of 14 (86%) PF samples (Table 4). Of these, 11 of 12 had only bacteria detected and 1 of 12 had viral-bacterial co-detection (*S. aureus* and human bocavirus) (Table 4). The predominant pathogen was *S. aureus* (7/14 [50%]) followed by *S. pneumoniae* (5/12 [36%]). Of 9 cases with culture and PCR results who had a pathogen identified, 5 (56%) had a pathogen identified by both tests (4/5 being *S. aureus*), and 4 (44%) had a pathogen identified by PCR alone (3/4 being *S. pneumoniae*) (Supplementary Table 5). Of the 12 PF-positive cases, 15 pathogens were detected on PF, of which 10 (67%) were also detected in either of the paired NP/OP or blood culture (Supplementary Table 3).

**Table 4.** Organisms Identified by Culture and/or Polymerase Chain Reaction of Pleural Fluid Specimens

Organism	PCR (n = 11)	Culture (n = 14)	Either PCR or Culture (n = 14)
Any positive^a^	9 (82)	9 (64)	12 (86)
* Streptococcus pneumoniae*	4 (36)	1 (7)	5 (36)
* Haemophilus influenzae*	1 (9)	0 (0)	1 (7)
* Staphylococcus aureus*	4 (36)	7 (50)	7 (50)
* Escherichia coli*	0	1 (7)	1 (7)
* Streptococcus* group F	0	1 (7)	1 (7)
* *HBOV	1 (9)	NA	1 (7)
Combinations^b^			
* S. aureus +* HBOV	…	…	1 (7)
* S. aureus + S. pneumoniae* ^c^	…	…	1 (7)
* E. coli* + *Streptococcus* group F + *H. influenzae*	…	…	1 (7)

Data are presented as no. (%).

Abbreviations: HBOV, human bocavirus; NA, not applicable; PCR, polymerase chain reaction.

^a^Total number of cases with organism identified; is not a sum of the number of organisms identified because some cases have >1 pathogen identified.

^b^Combinations presented as detection by either PCR or culture, not split by detection method.

^c^Of the 5 cases positive for *S. pneumoniae* in pleural fluid, 4 were identified by culture or PCR. One additional case was identified using BinaxNOW, which was also culture positive for *S. aureus* (see Supplemental Table 5). One *S. pneumoniae* PCR-positive case had a negative BinaxNOW test result.

Of the 5 PF cases positive for *S. pneumoniae*, 1 had a PCV13-type serotype detected (serotype 5) on culture from PF, blood, and NP (a 42-month-old child with 1 PCV dose) (Supplementary Table 6); the 4 remaining cases were positive by PCR or BinaxNOW only and not serotyped.

### Serious Adverse Events

There were 3 serious adverse events reported among LA cases (2 in-hospital deaths not clearly related to the procedure and 1 transient drop in oxygen saturation following the procedure), and none reported among PF cases (see details in the Supplementary Appendix). The 2 deaths in LA cases represented a case fatality rate (4.5%) that was no higher than among those with consolidation on CXR that did not have the procedure (8%) (odds ratio, 0.57 [95% confidence interval, .13–2.45]) (Table 1).

## DISCUSSION

We have described the range of pathogens identified by culture and PCR of lung and PF aspirates collected from African and Asian children with severe pneumonia and CXR consolidation in the PCV era. In LA, *S. pneumoniae* was the predominant pathogen identified, followed by *H. influenzae* and *M. catarrhalis,* whereas in PF *S. aureus* was the predominant pathogen, followed by *S. pneumoniae*. In comparison to culture alone, PCR increased the pathogen yield from 11% to 38% for LA (Table 2). From LA, *S. pneumoniae* was found as a single pathogen (5/9) as often as it was found in combination with other pathogens (4/9), usually other bacteria (3/9). In contrast, viruses were infrequently detected (n = 4), led by CMV (2/4).

Earlier pneumonia etiology studies conducted in the pre-PCV era that applied either standard culture, PCR, or both to lung aspirates reported *S. pneumoniae* as the predominant pathogen [4, 5, 20–22]. In this study, *S. pneumoniae* remained the leading pneumonia pathogen in the sampled group, despite the use of PCV in 3 of the 4 sites performing LA (all except Bangladesh). Viruses were identified infrequently in either LA or PF, in contrast to overarching PERCH etiology findings of 61.4% (95% credible interval, 57.3%–65.6%) of radiologically confirmed pneumonia attributed to viruses, with RSV the predominant virus identified [23]. Where viruses were identified in LA specimens, they were mostly detected in combination with bacteria (3/4), with no LA specimen positive for RSV. Also, despite the low prevalence of pleural effusions among all radiological pneumonias in this study (40 of 1935), the finding of *S. aureus* and *S. pneumoniae* as the leading pathogens in PF was similar to findings from other studies on pleural effusions, including studies in the post-PCV era [5, 24–30].

The difference in findings between the overall PERCH study, dominated by viral causes, and etiology of the LA cases, dominated by bacteria, is most likely because LA cases sampled represent a narrow clinical subgroup. Of note, LA cases came mostly from The Gambia, which had the second-highest etiology fraction for *S. pneumoniae* by site in the overall PERCH results (15.1%) [23]. Another consideration is the difficulty of attributing causality to the pathogens detected; while this appears less problematic for lung or pleural aspirates than for other samples, it is still an issue. The relative absence of viruses, for instance, may reflect the inability of even the best clinical samples taken at one point in time to elucidate causality due to a chain of pathogen and immune events. Nevertheless, the findings from the LA group do reinforce the possibility that polymicrobial disease is important [21]. Another possibility is that the PERCH study’s overall results underrepresented bacterial causes, although this risk was addressed by comprehensive methodologic and analytic efforts to account for the known difficulty in diagnosing bacterial pneumonia. With all these considerations in view, the findings do emphasize that even in the context of widespread PCV and Hib vaccination, and the increasing contribution of viral etiologies, bacterial infections remain important.

Having considered the differences of the findings of this study to those from all severe pneumonia cases in the PERCH study, we should also consider how generalizable the findings presented here are to children with consolidation on CXR. With the exception of site, demographic and clinical features were similar between those who underwent LA and those who did not, which is in favor of generalizability. Nevertheless, in the current study, only a small fraction (8%) of cases with CXR consolidation was sampled. Diagnostic yield, by either PCR or culture, for pneumococcus (20%) was lower than has been reported (25%–41%) in pneumonia etiology studies conducted in the pre-PCV era in similar settings [4, 5]. This lower diagnostic yield could be due to a number of factors including changing trends in antibiotic use and the impact of PCV, or may reflect sampling issues, bias from the small proportion of potentially eligible cases sampled, or chance. Rates of antibiotic use prior to LA collection appear considerably higher in PERCH (26/43 [60%]) than in a previous Gambian study (18%) [5]. The previous Gambian study was conducted in the pre-PCV era whereas PERCH was conducted after PCV introduction in all but 1 of the countries collecting LA samples, and we would expect to see a lower rate of detection of pneumococcus on LA specimens as a result. Nevertheless, in the current study, of the pneumococcal isolates for which serotype data were available, 67% (4/6) are included in current PCVs. Also, 39% of specimens were collected after the day of admission with the low yield (18%) in this group contributing to the lower-than-expected overall pathogen yield (Supplementary Table 4).

The outcomes of children who underwent the LA procedure in this study are consistent with the overall safety and utility of lung aspiration reported previously, with a serious adverse event rate no higher than in those who did not have the procedure. The procedure is not widely practiced among clinicians for various reasons, including unfamiliarity with the procedure and concerns regarding patient safety. One earlier report of >25 years’ experience of lung aspirations in The Gambia showed that there were infrequent episodes (<3%) of adverse events including minor bleeding and pneumothorax, all of which were transient [11]. The experience of the PERCH study is that clinicians in diverse settings were taught and successfully performed this procedure safely.

This study highlights the continued importance, even in the PCV era, of bacterial etiologies, prominently *S. pneumoniae* and *S. aureus*, in a select group of children with severe pneumonia associated with clear radiological consolidation or pleural effusion. Despite their limitations and lack of uptake, where appropriate skill and care are applied lung and pleural aspiration samples remain useful diagnostic specimens for such pneumonia cases, worthy of consideration both for research and clinical care. In this study they have provided an indication of possible differences in etiology between such cases and the majority of severe pneumonia cases, as demonstrated in the difference between the findings of this study and the viral etiology–dominated findings of the PERCH study overall. This study also highlights the value of molecular diagnostics for increasing the rate of pathogen detection.

## Supplementary Data

Supplementary materials are available at *Clinical Infectious Diseases* online. Consisting of data provided by the authors to benefit the reader, the posted materials are not copyedited and are the sole responsibility of the authors, so questions or comments should be addressed to the corresponding author.

ciaa1032_suppl_Supplementary_Tables

ciaa1032_suppl_Supplementary_Appendix

## Notes

***Acknowledgments.*** The authors offer sincere thanks to the patients and families who participated in this study. They are grateful to members of the Pneumonia Etiology Research for Child Health (PERCH) Study Group for conducting the studies and collecting data. They also recognize members of the PERCH chest radiograph reading panel and PERCH study team contributors for their roles in study conduct. This article was published with the permission of the Director of the Kenya Medical Research Institute.

***Financial support.*** This work was supported by the Bill & Melinda Gates Foundation (BMGF) (grant number 48968) to the International Vaccine Access Center, Department of International Health, Johns Hopkins Bloomberg School of Public Health; and the Wellcome Trust of Great Britain (clinical fellowship 098532) to J. A. G. S.

***Potential conflicts of interest.*** M. D. K. has received funding for consultancies from Merck, Pfizer, and Novartis, and grant funding from Merck. C. P. has received grant funding from Merck. K. L. O. has received grant funding from GlaxoSmithKline and Pfizer and participates on technical advisory boards for Merck, Sanofi-Pasteur, PATH, Affinivax, and ClearPath. K. L. K. has received grant funding from Merck Sharp & Dohme. W. A. B. has received funding from Sanofi, PATH, and BMGF, and contributions to contemporaneous studies from the Serum Institute of India, Roche, and Sanofi. L. L. H. reports grants to her institution from Merck, Pfizer, and MedImmune, outside the submitted work. S. C. M. reports grants to her institution from Wellcome Trust, outside the submitted work. N. F. reports consulting fees from Pfizer Inc, outside the submitted work. S. A. M. reports grants to his institution from Pfizer, Minervax, BMGF, and GSK, and advisory board fees from BMGF, outside the submitted work. All other authors report no potential conflicts of interest. All authors have submitted the ICMJE Form for Disclosure of Potential Conflicts of Interest. Conflicts that the editors consider relevant to the content of the manuscript have been disclosed.
