# Supplementary material for: The Etiology of Pneumonia From Analysis of Lung Aspirate and Pleural Fluid Samples: Findings From the Pneumonia Etiology Research for Child Health (PERCH) Study
Source: Clin Infect Dis. 2020 Jul 25;73(11):e3788–96. doi: 10.1093/cid/ciaa1032 (PMC8662778; doi:10.1093/cid/ciaa1032)
Supplement: ciaa1032_suppl_Supplementary_Tables [file ciaa1032_suppl_supplementary_tables.docx]

**Summary of Supplementary Materials:**

Supplemental Table 1. Clinical characteristics of cases with lung aspirates collected, by site

Supplementary Table 2. Comparison of clinical and demographic characteristics of cases with and without lung aspirates collected

Supplementary Table 3a. Comparison of blood culture, lower respiratory tract specimens and upper respiratory tract specimens for cases positive on lung aspirate

Supplementary Table 3b. Comparison of blood culture, lower respiratory tract specimens and upper respiratory tract specimens for cases positive on pleural fluid

Supplementary Table 4. Diagnostic yield by timing of lung aspirate collection

Supplemental Table 5a. Bacterial organisms identified by culture and/or PCR of lung aspirate specimens, among those with both culture and PCR results available

Supplemental Table 5b. Bacterial organisms identified in pleural fluid specimens with both culture and PCR test results available

Supplemental Table 6. Serotype results for cases culture positive for *S. pneumoniae* and *H. influenzae* on lung aspirate or pleural fluid

Supplemental Text: Serious Adverse Events

**Supplemental Table 1. Clinical characteristics of cases with lung aspirates collected, by site**

|  | **The Gambia (n=24)** | **Mali**  **(n=10)** | **South Africa (n=6)** | **Bangladesh (n=4)** |
| --- | --- | --- | --- | --- |
| **Age (months), median (IQR)** | 11 (4-23) | 4 (2-11) | 7 (4-9) | 18 (10.5-37) |
| **Age** |  |  |  |  |
|  | n (%) | n (%) | n (%) | n (%) |
| <1 year | 12 (50) | 8 (80) | 5 (83) | 1 (25) |
| ≥1 year | 12 (50) | 2 (20) | 1 (17) | 3 (75) |
| **Female** | 8 (33) | 6 (60) | 3 (50) | 3 (75) |
| **HIV positive** | 2 (8) | 1 (10) | 1 (17) | 0 (0) |
| **Pneumonia severity** |  |  |  |  |
| Severe | 18 (75) | 6 (60) | 4 (67) | 2 (50) |
| Very severe | 6 (25) | 4 (40) | 2 (33) | 2 (50) |
| **Hypoxaemic^a^** | 3 (12) | 6 (60) | 4 (67) | 1 (25) |
| **Tachycardia^b^** | 15 (62) | 8 (80) | 3 (50) | 0 (0) |
| **Duration of illness;**  **Median days (IQR)** | 3 (3-4.5) | 4.5 (3-7) | 4 (3-6) | 4.5 (2.5-6.5) |
| **Temperature >38°C** | 19 (79) | 1 (10) | 1 (17) | 1 (25) |
| **Wheeze^c^** | 3 (12) | 2 (20) | 0 (0) | 3 (75) |
| **Danger signs** |  |  |  |  |
| Head nodding | 1 (4) | 3 (30) | 2 (33) | 0 (0) |
| Central cyanosis | 1 (4) | 0 (0) | 0 (0) | 0 (0) |
| Inability to feed/ drink | 1 (4) | 0 (0) | 0 (0) | 1 (25) |
| Vomiting everything | 0 (0) | 0 (0) | 0 (0) | 0 (0) |
| Lethargy | 6 (25) | 1 (10) | 0 (0) | 1 (25) |
| Multiple or prolonged convulsions | 0 (0) | 1 (10) | 0 (0) | 2 (50) |
| **Antibiotic pre-treatment^d^** | 9 (38) | 8 (80) | 5 (83) | 4 (100) |
| **Vaccination** |  |  |  |  |
| At least 1 HibCV dose | 19 (86) | 8 (80) | 6 (100) | 4 (100) |
| At least 1 PCV dose | 18 (82) | 8 (80) | 5 (83) | 0 (0) |
| **Severe wasting^e^** | 0 (0) | 3 (30) | 1 (17) | 2 (50) |
| **Died in hospital** | 2 (8) | 0 (0) | 0 (0) | 0 (0) |

Abbreviations: LA, lung aspirate; PCV, pneumococcal conjugate vaccine; HibCV, *Haemophilus influenzae* type b conjugate vaccine.

Table restricted to sites where LA specimens were available. Cases with LA specimen collection more than 72 hours after enrollment were excluded from the analysis.

^a^ Hypoxaemic defined as oxygen saturation at admission <90% at South Africa and <92% at other sites, or oxygen requirement (if on oxygen and room air saturation not available).

^b^ Elevated heart rate at baseline clinical assessment defined as: greater than 160 bpm in infants 0-11 months, greater than 150 bpm in children 12-35 months, greater than 140 bpm in children 36-59 months.

^c^ Presence of audible or auscultatory wheeze at admission.

^d^ Antibiotic pretreatment was defined as having either a positive serum bioassay or documentation of antibiotics administered at the referral or study hospital prior to lung aspirate specimen collection.

^e^ Weight-for-height < -3 z-scores.

**Supplementary Table 2. Comparison of clinical and demographic characteristics of cases with and without lung aspirates collected**

|  | **A.**  **Cases with LA collected** | **B. Consolidation on CXR, no LA collected** | **C. Positive LA specimen** | **D. Negative LA specimen** | **p-value**  **(A vs. B)^a^** | **p-value**  **(C vs. D)^b^** | **p-value**  **(C vs. B)^b^** |
| --- | --- | --- | --- | --- | --- | --- | --- |
|  | n=44 | n=574 | n=13 | n=31 |  |  |  |
| **Age (months) Median (IQR)** | 8.5  (3-18.5) | 7 (3-13) | 9 (7-19) | 5 (3-18) | 0.18 | 0.17 | 0.05 |
| **Age <1 year** | 26 (59) | 396 (69) | 8 (62) | 18 (58) | 0.39 | 1 | 0.56 |
| **Female** | 20 (46) | 284 (50) | 3 (23) | 17 (55) | 0.69 | 0.10 | 0.09 |
| **Site** |  |  |  |  |  |  |  |
| The Gambia | 24 (54) | 81 (14) | 9 (69) | 15 (48) | <0.0001 | 0.009 | <0.0001 |
| Mali | 10 (23) | 135 (24) | 0 (0) | 10 (32) |  |  |  |
| South Africa | 6 (14) | 304 (53) | 4 (31) | 2 (7) |  |  |  |
| Bangladesh | 4 (9) | 54 (9) | 0 (0) | 4 (13) |  |  |  |
| **Very severe pneumonia (vs. severe)** | 14 (32) | 187 (33) | 6 (46) | 8 (26) | 0.27 | 0.29 | 0.37 |
| **Duration of illness;**  **Median days (IQR)^c^** | 3.5 (3-5.5) | 3 (2-6) | 4 (3-5) | 3 (3-6) | 0.90 | 0.78 | 0.76 |
| **Hypoxemic^d^** | 14 (32) | 321 (56) | 5 (38) | 9 (29) | 0.93 | 0.72 | 0.26 |
| **Tachycardia^e^** | 26 (59) | 293 (51) | 7 (54) | 19 (61) | 0.51 | 0.74 | 1 |
| **Temperature >38°C** | 22 (50) | 142 (25) | 9 (69) | 13 (42) | 0.30 | 0.19 | <0.0001 |
| **Wheeze^f^** | 8 (18) | 157 (27) | 0 (0) | 8 (26) | 0.11 | 0.08 | 0.02 |
| **Danger signs** |  |  |  |  |  |  |  |
| Head nodding | 6 (14) | 146 (25) | 2 (15) | 4 (13) | 0.80 | 1 | 0.53 |
| Central cyanosis | 1 (2) | 17 (3) | 1 (8) | 0 (0) | 0.77 | 0.3 | 0.34 |
| Inability to feed/drink | 2 (4) | 41 (7) | 1 (8) | 1 (3) | 0.86 | 0.51 | 1 |
| Vomiting everything | 0 (0) | 7 (1) | 0 (0) | 0 (0) | 0.73 | - | 1 |
| Lethargy | 8 (18) | 43 (8) | 4 (31) | 4 (13) | 0.07 | 0.21 | 0.02 |
| Multiple or prolonged convulsions | 3 (7) | 13 (2) | 0 (0) | 3 (10) | 0.14 | 0.54 | 1 |
| **Antibiotic pre-treatment^g^** | 11 (25) | 260 (45) | 3 (23) | 8 (26) | 0.34 | 1 | 0.19 |
| **Vaccination** |  |  |  |  |  |  |  |
| At least 1 HibCV dose | 35 (83) | 416 (77) | 13 (100) | 22 (76) | 0.98 | 0.08 | 0.08 |
| At least 1 PCV dose | 31 (74) | 355 (70) | 12 (92) | 19 (66) | 0.29 | 0.13 | 0.12 |
| **Severe wasting^h^** | 6 (14) | 100 (18) | 1 (8) | 5 (16) | 0.88 | 0.65 | 0.48 |
| **HIV positive** | 4 (9) | 69 (12) | 1 (8) | 3 (10) | 0.29 | 1 | 1 |
| **Died in hospital** | 2 (4) | 44 (8) | 1 (8) | 1 (3) | 0.48 | 0.51 | 1 |
| **RSV NP+** | 6 (14) | 129 (23) | 0 | 6 (20) | 0.85 | 0.16 | 0.08 |
| **Parainfluenza 1 NP+** | 3 (7) | 25 (4) | 1 (8) | 2 (7) | 0.50 | 1 | 0.43 |

Abbreviations: LA, lung aspirate; PCV, pneumococcal conjugate vaccine; Hib, *Haemophilus influenzae* type b conjugate vaccine.

Table restricted to sites where LA specimens were available (Gambia, South Africa, Bangladesh and Mali). Cases with LA specimen more than 72 hours after enrollment were excluded from the analysis.

1. P-values based on a logistic regression model adjusted for age in months and site, with Firth adjustment for categorical variables and Kruskal-Wallis test for continuous variables comparing cases with LA specimen taken to cases with consolidation on CXR without LA specimen taken.
2. P-values based on unadjusted logistic regression model, with Firth adjustment for categorical variables and Kruskal-Wallis test for continuous variables.
3. The number of days with cough, fever, difficulty breathing, wheeze, or runny nose, whichever symptom was longest.
4. Hypoxaemic defined as oxygen saturation at admission <90% at South Africa and <92% at other sites, or oxygen requirement (if on oxygen and room air saturation not available).
5. Elevated heart rate at baseline clinical assessment defined as: greater than 160 bpm in infants 0-11 months, greater than 150 bpm in children 12-35 months, greater than 140 bpm in children 36-59 months.
6. Presence of audible or auscultatory wheeze at admission.
7. Antibiotic pretreatment was defined as having either a positive serum bioassay or documentation of antibiotics administered at the referral or study hospital prior to NP/OP specimen collection.
8. Weight-for-height < -3 z-scores

**Supplementary Table 3a. Comparison of blood culture, lower respiratory tract specimens and upper respiratory tract specimens for cases positive on lung aspirate**

| **Case ID** | **Site** | **LA PCR** | **LA Culture** | **NP/OP PCR** | **Blood Culture** | **WB l*yt*A PCR**  **(for *S. pneumoniae*)** | **Age (m)** | **Duration of illness (days)** | **PCV doses** | **HIV status** | **Severe malnutrition** | **Prior antibiotic exposure** |
| --- | --- | --- | --- | --- | --- | --- | --- | --- | --- | --- | --- | --- |
| 1 | The Gambia | HIB  **HINF** | Negative | **HINF**^a^  MCAT  PNEU | **HINF** | Negative | 5 | 3 | 1 | Negative | No | No |
| 2 | The Gambia | **MCAT**  **PNEU** | **PNEU (ST 5)** | CMV  HBOV  HINF^a^  **MCAT**  **PNEU**^a^ **(ST 5)**  RHINO | Negative | Negative | 23 | 2 | 3 | Negative | No | No |
| 3 | The Gambia | **MCAT**  **PNEU** | **PNEU** (ST 1) | ADENO  CMV  HBOV  HINF^a^  **MCAT**  **PNEU**^a^ (19A) | Negative | Negative | 22 | 5 | 3 | Negative | No | No |
| 4 | The Gambia | **PNEU** | Negative | HINF  MCAT  **PNEU**  RHINO | Negative | Negative | 41 | 4 | 3 | Negative | No | No |
| 5 | The Gambia | **CMV** | Negative | **CMV**^a^  HINF^a^  MCAT  PNEU^a^ (ST 35B)  PV/EV | Negative | Negative | 7 | 4 | 3 | Negative | No | No |
| 6 | The Gambia | **PNEU** | Negative | COR43  HINF^a^  HMPV  MCAT  **PNEU**^a^ (ST 40) | Negative | Negative | 10 | 3 | 3 | Negative | No | No |
| 7 | The Gambia | Not available | **PNEU (ST 6A)** | HINF^a^  HMPV  MCAT  PARA1  **PNEU**^a^ (**ST 6A** and 19F)  PV/EV | Negative | Negative | 16 | 5 | 3 | Negative | No | No |
| 8 | The Gambia | Not available | **PNEU** (ST 12B) | CMV^a^  HINF^a^  MCAT  **PNEU**  PV/EV | **PNEU** (ST 12F) | Negative | 6 | 3 | 3 | Negative | No | No |
| 9 | The Gambia | HINF  CMV  MCAT  PJP  **PNEU** | HINF^b^ **PNEU** (ST 20) | Not available | Negative | **Positive^a^** | 9 | 6 | 3 | Negative | No | No |
| 10 | South Africa | **HINF**  **HMPV**  MCAT | Negative | CMV  **HINF**  **HMPV** | Negative | Negative | 6 | 2 | 2 | Negative | No | Yes |
| 11 | South Africa | **PNEU** | Negative | CMV^a^  HINF^a^  MCAT  **PNEU**^a^ (ST 15B/15C)^c^  RHINO  SAUR | Negative | **Positive^a^** | 8 | 4 | 2 | Positive | No | Yes |
| 12 | South Africa | **HINF**  **PNEU** | Negative | **HINF**^a^  MCAT  **PNEU**^a^ (ST 23F) | SASP | **Positive^a^** | 19 | 4 | 1 | Negative | Yes | Yes |
| 13 | South Africa | **ADENO**  **CPNEU** | Negative | **ADENO**  CMV  **CPNEU**  HINF^a^  FLUA  MCAT | Negative | Negative | 9 | 14 | 0 | Negative | No | No |

Severe malnutrition defined as weight-for-height < -3 z-scores. Bold indicates concordance in pathogens detected on lung aspirate (culture and/or PCR) and other specimens (NP/OP, blood culture, and/or whole blood PCR [*S. pneumoniae* only]).

During PERCH, PCV was in The Gambia (introduced August 2009), Mali (introduced March 2011), and South Africa (introduced April 2009).

Abbreviations: ADENO, adenovirus; CPNEU, *Chlamydophila pneumoniae*; CMV, human cytomegalovirus; COR43, coronavirus 43; CXR, chest x-ray; FLUA, influenza A; HBOV, human bocavirus; HIB, *Haemophilus influenzae* type b; HINF, *Haemophilus influenzae*; HIV, human immunodeficiency virus type 1; HMPV, human metapneumovirus; LA, lung aspirate; *lytA*, pneumolysin; MCAT, *Moraxella catarrhalis*; NP/OP, nasopharyngeal/oropharyngeal; PARA1, parainfluenza virus 1; PJP, *Pneumocystis jirovecii*; PCR, polymerase chain reaction; PNEU, *Streptococcus pneumoniae*; PV/EV, parechovirus/enterovirus; RHINO, human rhinovirus; SASP, *Salmonella* spp.; SAUR, *Staphylococcus aureus*; ST, serotype; WB, whole blood.

1. NP/OP PCR density above the threshold (6.9 log_10_ copies/ml for *S. pneumoniae*, 5.9 log_10_ copies/ml for *H. influenzae,* and 4.9 log_10_ copies/ml for CMV) or WB *lyt*A density above threshold (2.2 log_10_ copies/ml).
2. *H. influenzae* typing data not available from lung aspirate culture.
3. Serotype unresolved.

**Supplementary Table 3b. Comparison of blood culture, lower respiratory tract specimens and upper respiratory tract specimens for cases positive on pleural fluid**

| **Case ID** | **Site** | **PF PCR** | **PF Culture** | **PF PNEU BinaxNOW** | **NP/OP PCR** | **Blood Culture** | **WB l*yt*A PCR**  **(for *S. pneumoniae*)** | **Age (m)** | **PCV doses** | **HIV status** | **Severe malnutrition** | **Prior antibiotic exposure** |
| --- | --- | --- | --- | --- | --- | --- | --- | --- | --- | --- | --- | --- |
| 1 | The Gambia | Not available | SAUR | Not available | CMV^a^  COR43  C PNEU  HINF^a^  MCAT  PNEU^a^ (ST 6A) | Negative | Negative | 6 | 0 | Negative | No | No |
| 2 | Kenya | **PNEU** | **PNEU (ST 5)** | Positive | ADENO  COR63  HINF^a^  MCAT  **PNEU** | **PNEU (ST 5)** | **Positive^a^** | 42 | 1 | Negative | No | No |
| 3 | Kenya | Not available | **SAUR** | Not available | CPNEU  MCAT  PNEU (ST 16F)  PV/EV | **SAUR** | Negative | 7 | 3 | Negative | No | Yes |
| 4 | Mali | **SAUR** | **SAUR** | Not available | **SAUR** | Negative | Negative | 1 | 0 | Negative | No | No |
| 5 | Mali | **SAUR** | **SAUR** | Not available | HINF^a^  MCAT  PNEU^a^ (ST 38)  **SAUR** | Negative | Negative | 16 | 3 | Negative | No | No |
| 6 | Mali | **PNEU** | Negative | Not available | CMV  **PNEU**  PV/EV | Negative | Negative | 48 | 3 | Negative | No | Yes |
| 7 | Mali | **HINF** | ECOL STRF | Not available | CMV  **HINF** | Negative | Negative | 36 | 2 | Negative | No | No |
| 8 | South Africa | **PNEU** | Negative | **Positive** | CMV  HINF  MCAT  **PNEU** | Negative | **Positive^a^** | 18 | 2 | Negative | No | Yes |
| 9 | South Africa | **PNEU** | Negative | Negative | HINF^a^  MCAT  **PNEU** | Negative | **Positive^a^** | 35 | 3 | Negative | No | Yes |
| 10 | South Africa | SAUR | SAUR | Negative | HINF  MCAT  PNEU^a^ (ST 15B)  PV/EV | Negative | Negative | 48 | 2 | Negative | No | Yes |
| 11 | Zambia | Not available | **SAUR** | Positive | MCAT  PARA3  PNEU  **SAUR** | Negative | Not available | 13 | Not available | Negative | No | Yes |
| 12 | Zambia | HBOV **SAUR** | **SAUR** | Not available | CMV  HMPV  RHINO  **SAUR** | Negative | Negative | 3 | Not available | Negative | No | Yes |

Severe malnutrition defined as weight-for-height <-3 z-scores. Bold indicates concordance in pathogens detected on lung aspirate (culture and/or PCR) and other specimens (NP/OP, blood culture, and/or whole blood PCR [*S. pneumoniae* only]).

During PERCH, PCV was in routine use in The Gambia (introduced August 2009), Mali (introduced March 2011), and South Africa (introduced April 2009).

Abbreviations: ADENO, adenovirus; CMV, human cytomegalovirus; COR43, coronavirus 43; COR63, coronavirus 63; CPNEU, *Chlamydophila pneumoniae*; CXR, chest x-ray; ECOL, *Escherichia coli;* FLUA, influenza A; HBOV, human bocavirus; HINF, *Haemophilus influenzae*; HIV, human immunodeficiency virus type 1; HMPV, human metapneumovirus; MCAT, *Moraxella catarrhalis*; NP/OP, nasopharyngeal/oropharyngeal; PARA3, parainfluenza virus 3; PCR, polymerase chain reaction; PF, pleural fluid; PNEU, *Streptococcus pneumoniae*; PV/EV, parechovirus/enterovirus; RHINO, human rhinovirus; SASP, *Salmonella* spp.; SAUR, *Staphylococcus aureus*; ST, serotype; STRF, Streptococcus Group F.

1. NP/OP PCR density above the threshold (6.9 log_10_ copies/ml for *S. pneumoniae*, 5.9 log_10_ copies/ml for *H. influenzae,* and 4.9 log_10_ copies/ml for CMV) or WB *lyt*A density above threshold (2.2 log_10_ copies/ml).

**Supplementary Table 4. Diagnostic yield by timing of lung aspirate collection**

| Day lung aspirate collected | Number collected | Positive by lung aspirate PCR or culture n (%) |
| --- | --- | --- |
| Day of admission | 27 | 10 (37) |
| After admission | 17 | 3 (18) |
| Day 1 | 8 | 1 (13) |
| Day 2 | 8 | 1 (13) |
| Day 3 | 1 | 1 (100) |

Includes all cases with lung aspirate collected within 3 days of admission

**Supplemental Table 5a. Bacterial organisms identified by culture and/or PCR of lung aspirate specimens, among those with both culture and PCR results available**

|  | **Only detected on PCR (N=29) n (%)** | **Only detected on culture (N=29)**  **n (%)** | **Detected on both PCR and culture (N=29)**  **n (%)** |
| --- | --- | --- | --- |
| **Any positive^a^** | 6 (21) | 0 (0) | 3 (10) |
| ***S. pneumoniae*** | 3 (10) | 0 (0) | 3 (10) |
| ***H. influenzae*** | 3 (10) | 0 (0) | 1 (3) |
| ***M. catarrhalis*** | 4 (14) | 0 (0) | 0 (0) |
| ***C. pneumoniae*** | 1 (3) | 0 (0) | 0 (0) |

Table restricted to lung aspirate specimens with both culture and PCR results available, and bacterial organisms detected on PCR and/or culture.

a. Total number of cases with any positive identified is not the sum of the number of organisms identified because some cases had more than one pathogen identified.

**Supplemental Table 5b. Bacterial organisms identified in pleural fluid specimens with both culture and PCR test results available**

|  | **Only detected on PCR (N=11)**  **n (%)** | **Only detected on culture (N=11)**  **n (%)** | **Detected on both PCR and culture (N=11)**  **n (%)** |
| --- | --- | --- | --- |
| **Any positive^a^** | 4 (36) | 0 (0) | 5 (45) |
| ***S. pneumoniae*** | 3 (27) | 0 (0) | 1 (9) |
| ***H. influenzae*** | 1 (9) | 0 (0) | 0 (0) |
| ***S. aureus*** | 0 (0) | 0 (0) | 4 (36) |

Table restricted to pleural fluid specimens with both culture and PCR results available, and bacterial organisms detected on both PCR and culture. One case was positive for *H. influenzae* on pleural fluid PCR and culture positive for *E. coli* and Streptococcus Group F; for the purpose of this table this case was counted as PCR positive only.

a. Total number of cases with any positive identified is not the sum of the number of organisms identified because some cases have more than one pathogen identified.

**Supplemental Table 6. Serotype results for cases culture positive for *S. pneumoniae* and *H. influenzae* on lung aspirate or pleural fluid**

| **Case ID** | **Site** | **LA Culture** | **PF Culture** | **NP Culture** | **Blood Culture** |
| --- | --- | --- | --- | --- | --- |
| 1 | The Gambia | *S. pneumoniae* (ST 5) |  | *S. pneumoniae* (ST 5) | Negative |
| 2 | The Gambia | *S. pneumoniae* (ST 1) |  | *S. pneumoniae* (ST 19A) | Negative |
| 3 | The Gambia | *S. pneumoniae* (ST 6A) |  | *S. pneumoniae* (ST 19F and 6A) | Negative |
| 4 | The Gambia | *S. pneumoniae* (ST 12F) |  | *S. pneumoniae* (ST 12F)^a^ | *S. pneumoniae*  (ST 12F) |
| 5 | The Gambia | *S. pneumoniae* (ST 20)  *H. influenzae* (N/A) |  | Missing | Negative |
| 6 | Kenya |  | *S. pneumoniae*  (ST 5) | *S. pneumoniae* (ST 5)^a^ | *S. pneumoniae*  (ST 5) |

Abbreviation: LA, lung aspirate; N/A, not available; NT, non-typeable; NP, nasopharyngeal; PF, pleural fluid; ST, serotype.

^a^ *S. pneumoniae* detected by culture but NP PCR density below threshold. Neither case had evidence of antibiotics prior to NP/OP collection.

**Supplemental Text: Serious Adverse Events**

There were three serious adverse events (SAEs) reported among LA cases (two from The Gambia and one from Bangladesh): two in-hospital deaths occurred, and one case experienced a transient drop in oxygen saturation below 92% following the procedure. There were no SAEs reported among cases who underwent a pleural fluid aspiration.

One death was in a 9-month old male with very severe pneumonia. This infant was very ill prior to the procedure, was initially stabilized prior to the LA procedure but continued to deteriorate post-LA procedure and died within two hours of the procedure. The independent safety monitor judged that this death was unrelated to the procedure, while the Johns Hopkins School of Public Health Institutional Review Board categorized it as possibly related on the basis of the temporal sequence alone. The other death was in a 4-month old male who was admitted with a diagnosis of sepsis, had the LA procedure done 11 days after admission but succumbed to his illness 10 days later. The episode of transient desaturation, resolved within 1 hour of the initial desaturation event, was reported in a 5-month old male.
