# Supplementary material for: The Etiology of Pneumonia From Analysis of Lung Aspirate and Pleural Fluid Samples: Findings From the Pneumonia Etiology Research for Child Health (PERCH) Study
Source: Clin Infect Dis. 2020 Jul 25;73(11):e3788–96. doi: 10.1093/cid/ciaa1032 (PMC8662778; doi:10.1093/cid/ciaa1032)
Supplement: ciaa1032_suppl_Supplementary_Appendix [file ciaa1032_suppl_supplementary_appendix.docx]

**Pleural fluid findings and review process**

The PERCH Chest Radiograph Reading Panel did not standardize or adjudicate on pleural effusion. We observed considerable discordance between CXR readers with regards to presence of pleural effusion and did not have final data on pleural effusion at the case level. Across all 3973 images, there were 242 images where any one of the readers or arbitrators indicated pleural fluid on CXR and 60 images where at least two readers or arbiters indicated pleural fluid on CXR. Restricting to the CXR readers, there were 200 images where pleural fluid was identified by one of the two readers. Of those 200 images, only 38 images had concordance between readers (19%), where both indicated pleural fluid.

In order to define the subset of the cases with pleural effusion on CXR, clinicians from The Gambia PERCH site reviewed all of the CXRs from those cases where two or more standardized readers indicated pleural effusion, or the case had a pleural fluid specimen obtained. The cases confirmed to have presence of any pleural effusion on CXR by the clinical review team were included in the analysis and classified based on if they had a pleural fluid specimen obtained within 3 days of admission or had no pleural fluid specimen obtained (Table 2).

**Analytic differences with main PERCH descriptive and aetiology papers**

This analysis had several key differences from the main PERCH descriptive and etiology papers. First, the main PERCH papers were restricted to HIV-uninfected study participants. This analysis included HIV-infected PERCH cases as well. Second, the main PERCH papers did not incorporate the results of the CXR pleural effusion review by clinicians from The Gambia PERCH site. Lastly, the laboratory results reported in the main paper took into account findings from a clinical adjudication process. This process involved clinicians reviewing culture and PCR results in the context of clinical and other laboratory results in order to determine whether the organism detected was the likely cause of hospitalization or a contaminant. This current analysis reports the culture and PCR results regardless of the clinical adjudication findings, including two organisms detected on LA PCR (CMV and *P. jirovecii*) and one organism detected on PF PCR (*H. influenzae*) that the clinical adjudication process removed in the main paper results.

**Eligibility criteria for lung aspirate and pleural fluid collection**

Lung aspirate collection was performed at four sites (The Gambia, Bangladesh, Mali, and South Africa). Eligible cases were those with large, dense peripheral consolidation on CXR. Contraindications for lung aspirate collection included:

1. Presence of pneumatocoeles on CXR.
2. Post measles pneumonia.
3. If the patient was clinically unstable as determined by a clinician, the procedure would be deferred until stabilisation.
4. Cardiorespiratory resuscitation (CPR) performed within the last 24 hours.
5. Parental refusal to have their child subjected to lung aspiration.

Pleural fluid was collected from a minority of cases as indicated by attending clinicians. The methodology for obtaining pleural fluid followed local clinical practice guidelines, including standard safety precautions. Contraindications for pleural fluid collection included:

1. Coagulopathy or thrombocytopenia.
2. Haemodynamic or respiratory instability (unless therapeutic thoracentesis was required for management of clinical instability).

**PCR testing**

The Fast-track Diagnostics Respiratory Pathogens 33 (FTD Resp-33 kit; Fast-track Diagnostics, Sliema, Malta) multiplex PCR kit which was used in PERCH includes the following 33 viral, bacterial and fungal targets:

- influenza A, B and C
- parainfluenza viruses types 1, 2, 3 and 4
- coronaviruses NL63, 229E OC43 and HKU1
- human metapneumovirus A/B
- human rhinovirus
- respiratory syncytial virus A/B
- adenovirus
- enterovirus
- parechovirus
- bocavirus
- cytomegalovirus
- *Pneumocystis jirovecii*
- *Mycoplasma pneumoniae*
- *Chlamydophila pneumoniae*
- *Streptococcus pneumoniae*
- *Haemophilus influenzae* type b
- *Haemophilus influenzae* species
- *Staphylococcus aureus*
- *Moraxella catarrhalis*
- *Bordetella pertussis*
- *Klebsiella pneumoniae*
- Legionella species
- Salmonella species

## **Pneumococcal Serotyping**

Pneumococcal serotypes were determined from culture isolates by Quellung and/or PCR, or by microarray (NP/OP only) for specimens that were NP-culture negative and PCR-positive.^1^

1 Driscoll AJ, Karron RA, Morpeth SC, *et al.* Standardization of Laboratory Methods for the PERCH Study. *Clin Infect Dis* 2017; **64**: S245–52.
